# *Ab initio* study on the excited states of pyrene and its derivatives using multi-reference perturbation theory methods†

**DOI:** 10.1039/c9ra10483f

**Published:** 2020-03-31

**Authors:** Soichi Shirai, Shinji Inagaki

**Affiliations:** Toyota Central R&D Laboratories, Inc. Nagakute Aichi 480-1192 Japan shirai@mosk.tytlabs.co.jp

## Abstract

Low-lying singlet excited states of pyrene derivatives originated from the ^1^L_a_ and ^1^L_b_ states of pyrene have decisive influences on their absorption and fluorescence emission behaviors. Calculation of these excited states with quantitative accuracy is required for the theoretical design of pyrene derivatives tailored to target applications; this has been a long-standing challenge for *ab initio* quantum chemical calculations. In this study, we explore an adequate computational scheme through calculations of pyrene and its phenyl-substituted derivatives using multi-reference perturbation theory (MRPT) methods. All valence π orbitals on the pyrene moiety were assigned to the active orbitals. Computational load was reduced by restricting the electron excitations within the active orbitals in the preparation of reference configuration space. A generalized multi-configuration quasi-degenerate perturbation theory (GMCQDPT) was adopted to treat the reference space other than the complete active space. The calculated ^1^L_a_ and ^1^L_b_ excitation energies of pyrene are in good agreement with the experimental values. Calculations of 1,3,6,8-tetraphenyl pyrene suggest that the energetic ordering of ^1^L_a_ and ^1^L_b_ is inverted through tetraphenyl substitution and its lowest singlet excited state is the ^1^L_a_ parentage of pyrene, which is consistent with the experimentally deduced scheme. These results are not readily obtained by MRPT calculations with a limited number of active orbitals and single-reference theory calculations. Diphenyl pyrenes (DPPy) were also calculated at the same level of theory to investigate the dependence on the substitution positions of phenyl groups.

## Abstract 2

The excited states of phenyl-substituted pyrene derivatives were calculated using multi-reference perturbation theory methods.

## Graphical abstract

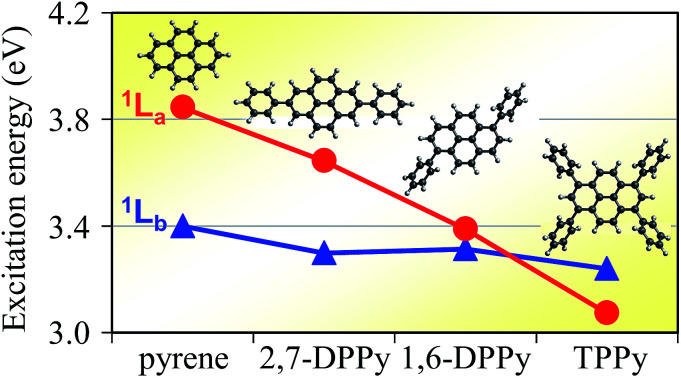

## 1. Introduction

Pyrene is one of the most well-studied polycyclic aromatic hydrocarbons (PAHs) because of its characteristic photophysical properties, such as prominent absorption bands and a fluorescence emission.^1^ These properties can be tailored to meet specific requirements through modifications of its chemical structure;^2–11^ therefore, pyrene derivatives are recognized as promising materials for organic light-emitting devices.^12–25^ Pyrene is also well known for the formation of an excimer.^26–29^ An aromatic excimer is a dimeric complex of the same aromatic molecules that is formed in the excited state. The fluorescence emission of an excimer was firstly observed for a pyrene solution by Förster and Kasper in 1954.^27^ The excimer fluorescence emission band is significantly red-shifted, broad, and structureless, so that it is clearly distinguishable from that of the monomer. Given the substantial distinction between monomer and excimer emissions, as well as their sensitivity to the surrounding environments, pyrene is used to study the molecular structural properties of polymers^30–34^ and macromolecules, such as proteins^35,36^ and DNA.^37–42^ It is also used as a probe in chemosensors that detect particular metal ions^43–51^ and molecules.^52–55^ The properties of an excimer are closely related to those of the monomer.^56–58^ Thus, the excited states of pyrene and its derivatives have been vigorously investigated and still continue to attract much interest from scientists with respect to both fundamental studies and practical applications.^59–64^

Pyrene has two important excited states, ^1^L_a_ and ^1^L_b_ in Platt's notation.^65^ These excited states are closely relevant to the absorption and fluorescence emission behavior of pyrene. Excitation from the ground state to the ^1^L_a_ state gives a prominent absorption band around 340 nm in the UV/Vis spectrum because of its large oscillator strength. In contrast, the oscillator strength of the ^1^L_b_ state is negligibly small; the ^1^L_b_ absorption band is barely visible in the absorption spectrum.^66^ The ^1^L_b_ state is the lowest singlet excited state and the ^1^L_a_ state is the second lowest; therefore, the fluorescence emission of pyrene is of ^1^L_b_ parentage according to Kasha's rule.^67^ In substituted pyrene derivatives, the excitation energies and absorbance of these excited states are perturbed by the substituents. In some cases, even an energetic ordering of the ^1^L_a_ and ^1^L_b_ states is inverted as a result of the energy-level shifts of these excited states. 1,3,6,8-Tetraphyenyl pyrene (TPPy) is recognized as a typical example.^68,69^ TPPy exhibits a relatively short fluorescence lifetime (*τ* ≈ 3 ns)^68,69^ and a high fluorescence quantum yield (*q*_F_ = 0.9)^70^ compared to pyrene (*τ* > 300 ns and *q*_F_ = 0.32).^68,69,71^ These conspicuous changes in the photophysical properties can be attributed to an inversion of the energetic ordering of the ^1^L_a_ and ^1^L_b_ states through tetraphenyl substitution. Assuming the ^1^L_a_–^1^L_b_ inversion, the fluorescence emission from TPPy originates from the ^1^L_a_-derived excited state of pyrene with a large oscillator strength, which is consistent with the experimental results.^68–70^ Although this hypothetical mechanism based on the experimental findings was supported by theoretical studies that employed semi-empirical methods^68,69,72^ and has been widely accepted, the ^1^L_a_–^1^L_b_ inversion in TPPy still remains to be corroborated by *ab initio* calculations. Given such a background, a dependable computational scheme that enables accurate calculations of these excited states is required for the theoretical design of pyrene derivatives customized to target applications; therefore, the prediction of their energetic ordering is of particular importance.

Calculation of the ^1^L_a_ and ^1^L_b_ excited states of pyrene with quantitative accuracy has been a long-term challenge for *ab initio* quantum chemistry.^73^ Time-dependent density functional theory (TDDFT)^74–78^ has been recognized as an efficient approach to the excited states of large molecules^79,80^ and is thus utilized in investigations of pyrene and its derivatives. Behind numerous successful results, comprehensive assessments indicate that TDDFT calculations yield inconsistencies in accuracy between the ^1^L_a_ and ^1^L_b_ excitation energies; in the worse cases, even the energetic ordering is incorrectly predicted.^73,81,82^ Similar problems are known for TDDFT calculations of some other PAHs.^83–90^ The second-order approximate coupled cluster singles and doubles (CC2) method is also widely used in the calculation of large molecules as an *ab initio* wave function approach.^91^ The energetic ordering of ^1^L_a_ and ^1^L_b_ of pyrene given by CC2 calculation is generally consistent with the experimental results. However, the incorporation of multi-configuration characters of excited states using single-reference methods such as CC2 is possibly insufficient, and could thus be responsible for the calculation errors. In this regard, multi-reference methods are expected to make up for this shortcoming.^92^ Bito *et al.* conducted multi-reference configuration interaction (MRCI) calculations as pioneering work,^93^ followed by theoretical studies that utilized multi-reference perturbation theory (MRPT) methods,^73,81,94^ such as multi-configuration quasi-degenerate perturbation theory (MCQDPT)^95^ and complete active space second-order perturbation theory (CASPT2).^96^ These methods adopt a complete active space self-consistent field (CASSCF) wavefunction^97^ as their reference. The CAS involves all electron configurations generated by distributing active electrons among active orbitals. Consequently, the dimension rapidly increases with the number of active orbitals and electrons, which makes the routine computation impossible. Therefore, in these studies, a limited number of π orbitals and π electrons were selected from a total of 16 valence π orbitals and 16 valence π electrons to construct the reference CAS. Although the energetic ordering of ^1^L_a_ and ^1^L_b_ is correctly predicted in most of these calculations, absolute errors in the excitation energies of 0.2–0.7 eV remain, and the calculation accuracy is inconsistent between the ^1^L_a_ and ^1^L_b_ states. The discrepancies at the CASSCF level are much larger because dynamical electron correlation is not sufficiently incorporated.

Recent advances in theory and computational techniques have yielded significant progress. Freidzon *et al.* demonstrated that the ^1^L_a_ and ^1^L_b_ excitation energies of pyrene can be accurately predicted^98^ using extended MCQDPT (XMCQDPT) method.^99^ Nenov *et al.* performed second-order perturbation theory restricted active space (RASPT2) calculations^100^ with the reference configuration space constructed using 16 π orbitals and 16 π electrons.^101^ The calculated excitation energies were in good agreement with the experimental values, even though the computational load was reduced by restricting the electron excitations within the active orbitals. Most recently, Noble *et al.* conducted multi-state CASPT2 calculations with the Cholesky decomposition technique^102,103^ in their study of electronic relaxations from the S_3_ state of pyrene.^104^ The reference configuration space was prepared using 16 π orbitals and 16 π electrons, and the results were quite accurate. Lischka *et al.* carried out the calculations of paradigmatic aromatic molecules including pyrene using both multi-reference and single-reference methods.^105^ In their results, the ^1^L_a_ and ^1^L_b_ excitation energies of pyrene calculated using the DFT/MRCI method^106^ were in good agreement with the experimental values. The same authors successfully applied their calculation scheme to the study of large-sized aromatic dimers.^107^ These studies suggest that the excited states of pyrene derivatives could also be calculated with quantitative accuracy by utilizing these advanced methods. Successful results with the full π valence reference space also imply that the incorporation of multi-configuration character is of key importance for accurate calculation.

**Fig. 1:**
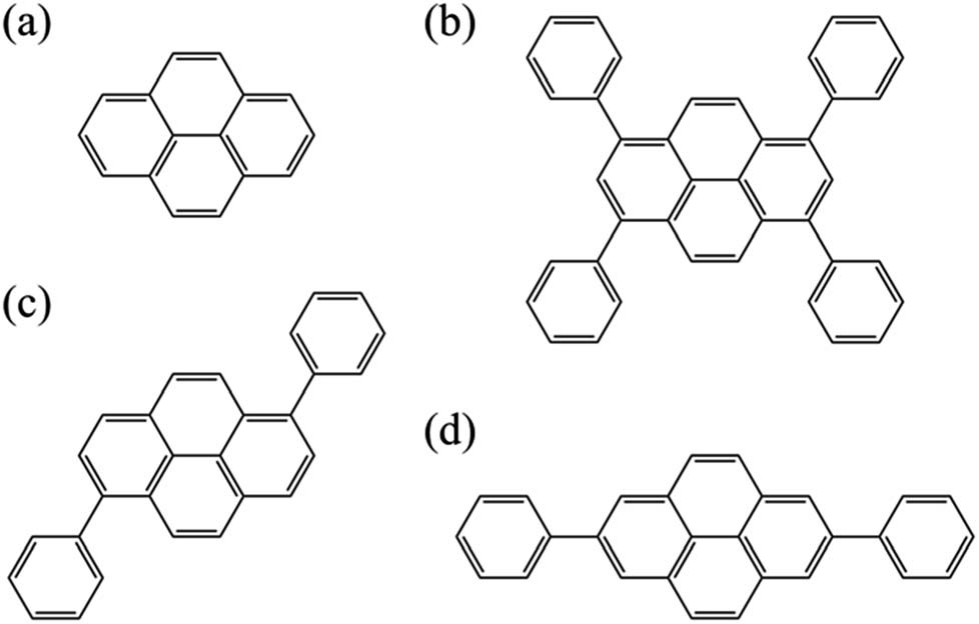
Molecular structures of (a) pyrene, (b) 1,3,6,8-tetraphenylpyrene (TPPy), (c) 1,6-diphenylpyrene (1,6-DPPy), and (d) 2,7-diphenylpyrene (2,7-DPPy).

In this study, we explore an adequate computational scheme that is suitable for the excited states of pyrene derivatives through calculations of pyrene and its phenyl substituted-derivatives shown in Fig. 1. Generalized multi-configuration quasi-degenerate perturbation theory (GMCQDPT)^108^ was adopted in addition to the original MCQDPT. In contrast to the conventional multi-reference theories where the reference space is limited to the CAS, GMCQDPT allows more general types of reference. Accordingly, it has the potential to reduce the computational load without a significant decrease of accuracy and could enable the handling of pyrene derivatives with large system sizes.

## 2. Computational details

### 2.1. Reference wave functions for GMCQDPT

In the preparation of the reference wave function for GMCQDPT, the molecular orbitals are divided into three groups: inactive, active, and external orbitals. Whereas the inactive orbitals are always doubly occupied, external orbitals are always vacant. The electrons involved in the active orbitals are regarded as active electrons and the electron configurations are generated by distributing active electrons among active orbitals. The procedure so far is the same as that adopted in the construction of the CAS. Although any types of reference configuration space are available in GMCQDPT, the following three are proposed as typical examples: multi-reference determinant list plus excitations (MRX), restricted active space (RAS), and occupation restricted multiple active space (ORMAS).^109–111^ In this study, the MRX type of reference was employed because of its simplicity. In the MRX framework, parent configurations and the electron excitation level, *n*, are defined. The excited configurations having *n* or less excited electrons from the parent configurations are involved into the reference space, in addition to the parent configurations themselves. If *n* is equal to the number of active electrons, then the reference space is identical with the CAS. The dimension of the reference space can be reduced by limiting the number of parent configurations and specifying *n* less than the number of active electrons.

In the GMCQDPT calculations of pyrene in this study, all 16 valence π orbitals were selected as active orbitals. Similarly, in the calculations of phenyl-substituted derivatives, the 16 π orbitals on their pyrene moieties were treated as active orbitals. The Hartree–Fock type ground state configuration with eight doubly-occupied π orbitals and eight unoccupied π* orbitals was defined as a parent configuration, and the *n* value was set at 2, 3 and 4. Henceforth, these reference spaces are denoted as MRX(*n*). GMCQDPT calculations were performed in the following two steps as well as the conventional CASSCF–MCQDPT procedure: multi-configuration self-consistent field (MCSCF) calculation with MRX(*n*) configuration space was firstly performed; the second-order perturbation calculation was subsequently conducted employing the obtained MCSCF function as its reference.

### 2.2. Calculations

The molecular geometrical structures were optimized using DFT with the B3LYP functional.^112,113^ The ground and excited states were subsequently calculated at the optimized geometries. The absence of an imaginary number frequency was confirmed for all optimized structures by vibrational analyses. In addition to the GMCQDPT calculations, conventional CASSCF–MCQDPT calculations were also performed for comparison; CAS(4πe, 4πo), CAS(8πe, 8πo), and CAS(12πe, 12πo) were used as reference spaces. CAS{(2*m*)πe, (2*m*)πo} (*m* = 2, 4, and 6) was constructed using the *m* highest occupied and the *m* lowest unoccupied π orbitals on the pyrene moiety. The ground state, and the ^1^L_a_ and ^1^L_b_ excited states were averaged with even weights in the MCSCF calculations, and these three states were simultaneously perturbed in the MRPT calculations. Equations of motion coupled cluster singles and doubles (EOM-CCSD),^114,115^ and TDDFT calculations with B3LYP, CAM-B3LYP,^116^ and ωB97XD^117^ functionals were also performed. The DPPy derivatives were calculated using GMCQDPT with MRX(4) and TDDFT. The cc-pVDZ basis set was used throughout the calculations.^118^ The molecular symmetries assumed were *D*_2h_, *D*_2_, *C*_2_, and *C*_2h_ for pyrene,^119^ TPPy,^120^ 1,6-DPPy, and 2,7-DPPy,^121^ respectively. MCSCF and MRPT calculations were performed using the GAMESS program.^122,123^ Other calculations were conducted using Gaussian09.^124^

## 3. Results and discussion

### 3.1. Calculation results

**Fig. 2:**
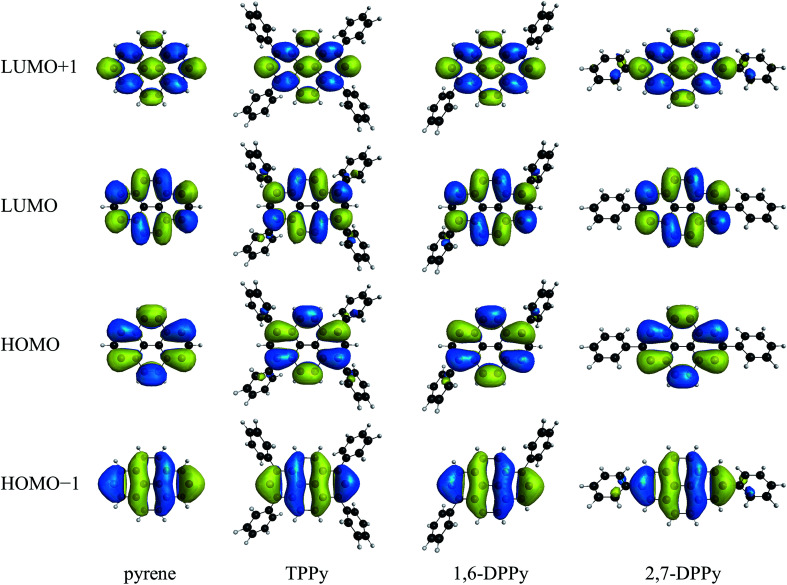
Natural orbitals of pyrene, TPPy, 1,6-DPPy, and 2,7-DPPy from the MCSCF wave functions with the MRX(4) configuration space.

The calculated excitation energies and oscillator strengths are summarized in Table 1 along with available experimental values.^119–121,125–128^ According to the previous studies, the ^1^L_a_ state of pyrene is characterized by two singly excited configurations: one is the highest occupied molecular orbital (HOMO) → the lowest unoccupied molecular orbital (LUMO) single excitation, and the other is the HOMO−1 → LUMO+1 single excitation. In contrast, HOMO−1 → LUMO and HOMO → LUMO+1 single excitations are dominant in the ^1^L_b_ state.^119,129^ The excited states were then identified on the basis of these configurations and related molecular orbitals. Fig. 2 shows the natural orbitals from HOMO−1 to LUMO+1 obtained using MCSCF with the MRX(4) configuration space. All natural orbitals from the MCSCF wave functions are presented in Fig. S1–S3.† Electronic state total energies and MCSCF excitation energies are listed in Table S1.†

**Table 1:** Calculated excitation energies and oscillator strengths. Available experimental values are also presented

Molecule	Method	Reference space	Number of electron configurations	^1^L_a_		^1^L_b_	
				Excitation energy (eV)	Oscillator strength^a^	Excitation energy (eV)	Oscillator strength^a^
Pyrene	GMCQDPT	MRX(2)	4349	3.75	0.2030	3.17	0.0000
		MRX(3)	84 317	3.82	0.2511	3.31	0.0000
		MRX(4)	853 785	3.84	0.2308	3.40	0.0000
	MCQDPT	CAS(4πe, 4πo)	28	3.55	0.2170	2.83	0.0007
		CAS(8πe, 8πo)	3684	3.75	0.3997	3.26	0.0000
		CAS(12πe, 12πo)	640 432	3.83	0.3668	4.01	0.0001
	EOM-CCSD			4.45	0.3443	3.82	0.0001
	TD-B3LYP			3.68	0.2534	3.74	0.0001
	TD-CAM-B3LYP			3.98	0.3169	3.95	0.0001
	TD-ωB97XD			3.99	0.3216	3.96	0.0001
	Exptl.			3.85^b^, 3.87^c^, 3.71^d^		3.41^b^, 3.37^c^^,^^e^	
							
TPPy	GMCQDPT	MRX(2)	4349	3.11	0.2748	2.96	0.0000
		MRX(3)	84 317	3.27	0.3607	3.12	0.0001
		MRX(4)	853 785	3.07	0.2853	3.24	0.0000
	MCQDPT	CAS(4πe, 4πo)	28	2.92	0.3832	2.60	0.0028
		CAS(8πe, 8πo)	3684	3.15	0.5360	3.04	0.0003
		CAS(12πe, 12πo)	640 432	3.69	0.5381	3.58	0.0000
	EOM-CCSD			3.91	0.9563	3.65	0.0001
	TD-B3LYP			3.13	0.7128	3.49	0.0003
	TD-CAM-B3LYP			3.47	0.8555	3.76	0.0008
	TD-ωB97XD			3.50	0.8649	3.77	0.0007
	Exptl.			3.24^f^, 3.15^g^			
							
1,6-DPPy	GMCQDPT	MRX(4)	1 138 281	3.39	0.2438	3.31	0.0169
	TD-B3LYP			3.35	0.6050	3.61	0.0025
	TD-CAM-B3LYP			3.69	0.7010	3.85	0.0085
	TD-ωB97XD			3.71	0.7009	3.86	0.0093
							
2,7-DPPy	GMCQDPT	MRX(4)	853 785	3.64	0.1487	3.30	0.0001
	TD-B3LYP			3.59	0.1276	3.38	0.0000
	TD-CAM-B3LYP			3.92	0.2198	3.71	0.0000
	TD-ωB97XD			3.94	0.2544	3.74	0.0000
	Exptl.			3.63^h^			

a Values are rounded off to four decimal places.

b Ref. 125; adiabatic transition energies in vapor.

c Ref. 126; gas-phase fluorescence excitation spectrum.

d Ref. 127; *λ*_max_ in acetonitrile.

e Ref. 119; gas-phase fluorescence excitation spectrum.

f Ref. 128; *λ*_max_ in CH_2_Cl_2_.

g Ref. 120; *λ*_max_ in CH_2_Cl_2_.

h Ref. 121; *λ*_max_ in dilute CH_2_Cl_2_ (*ca.* 1 × 10^−5^ M).

#### 3.1.1. Pyrene

The GMCQDPT-calculated excitation energies of pyrene exhibit systematic improvements with increasing *n*. The calculated ^1^L_b_ excitation energy with MRX(2) is underestimated by approximately 0.2 eV; the deviation is suppressed with an increase of the *n* value; the ^1^L_a_ and ^1^L_b_ excitation energies calculated with MRX(3) and MRX(4) are both in good agreement with the experimental values. In contrast, the MCQDPT results largely fluctuate depending on the reference space. Both the ^1^L_a_ and ^1^L_b_ excitation energies with CAS(8πe, 8πo) are relatively close to the corresponding experimental values, whereas both values are significantly underestimated with CAS(4πe, 4πo). The ^1^L_a_ excitation energy calculated with CAS(12πe, 12πo) is close to the experimental values; however, the ^1^L_b_ excitation energy is overestimated by as much as *ca.* 0.6 eV, so that the predicted energetic ordering of these two states is contrary to the experimental results. The ^1^L_b_ excitation energy is more sensitive to the reference space than the ^1^L_a_ excitation energy, which suggests its considerable multi-configuration character. The EOM-CCSD calculation overestimates the excitation energies by approximately 0.5 eV for both ^1^L_a_ and ^1^L_b_. The calculation results with the TDDFT method are similar to those reported in pioneering works.^73,81,82,105,130,131^ In the B3LYP results, the ^1^L_a_ excitation energy is underestimated, whereas the ^1^L_b_ excitation energy is overestimated. The predicted ordering of these states in energy is consequently inconsistent with the experimental ordering. Although the calculated ordering is marginally correct in the CAM-B3LYP and ωB97XD results, the excitation energies of the ^1^L_a_ and ^1^L_b_ states are quite close to each other due to serious overestimations of the ^1^L_b_ energy level. Thus, reliable calculations of the ^1^L_a_ and ^1^L_b_ excitation energies could not be accomplished using the exchange-correlation functionals examined here. The calculated oscillator strengths of the ^1^L_a_ state are much larger than those of the ^1^L_b_ state in all cases, which is in agreement with the experimental observations.^66^

#### 3.1.2. TPPy

The excitation energies calculated for TPPy are generally decreased from those for pyrene. The GMCQDPT calculations suggest that the ^1^L_a_ excitation energy decreases to a greater extent than the ^1^L_b_ excitation energy from that of pyrene through tetraphenyl substitution. As a result, the ^1^L_a_ energy level approaches ^1^L_b_ in the results with MRX(2) and MRX(3). However, the ^1^L_a_ state is still higher-lying than the ^1^L_b_ state in these results. In contrast, the energetic ordering of ^1^L_a_ and ^1^L_b_ is inverted in the calculation results with MRX(4), and the ^1^L_a_ state is determined to be the lowest singlet excited state.^68,69^ In addition, the ^1^L_a_ excitation energies calculated with GMCQDPT are close to the experimental value. The MCQDPT results largely vary depending on the reference space, similar to the calculations for pyrene. The results with CAS(4πe, 4πo) and CAS(8πe, 8πo) are similar to the GMCQDPT calculations with MRX(2) and MRX(3): the ^1^L_a_ state approaches ^1^L_b_ state in terms of energy; however, it is still higher-lying than the ^1^L_b_ state and the ^1^L_a_–^1^L_b_ inversion is not predicted. The calculations with CAS(12πe, 12πo) give confusing results: the incorrectly-predicted energetic ordering of ^1^L_a_ and ^1^L_b_ for pyrene is inverted, which results in another inconsistency with the experimentally-deduced scheme. The EOM-CCSD calculation fails to predict the ^1^L_a_–^1^L_b_ inversion through tetraphenyl substitution and overestimates the ^1^L_a_ excitation energy. Given the overestimations of the excitation energies in pyrene, the ^1^L_b_ excitation energy of TPPy may also be overestimated. The TDDFT calculations predict that the ^1^L_a_ state is lower-lying than the ^1^L_b_ state in TPPy, which is similar to the GMCQDPT calculations with MRX(4). The ^1^L_a_ excitation energy with B3LYP is in good agreement with the experimental value, whereas CAM-B3LYP and ωB97XD give overestimated values. The ^1^L_b_ excitation energies are higher than that obtained using GMCQDPT with MRX(4) by 0.25 eV for B3LYP and by *ca.* 0.5 eV for the other two functionals.

Overall, provided that the GMCQDPT method along with MRX(4) is adopted, the excitation energies of pyrene and TPPy can be accurately calculated, and the ^1^L_a_–^1^L_b_ inversion through tetraphenyl substitution is predicted. The calculated oscillator strength of the ^1^L_a_ state for TPPy is consistently larger than that of pyrene, whereas that of the ^1^L_b_ state is still vanishingly small.

#### 3.1.3. 1,6-DPPy

The calculations suggest that the excitation energies of the DPPy derivatives are generally lower than those of pyrene. Let us first examine the calculation results of 1,6-DPPy. In the calculation results of GMCQDPT with MRX(4), the ^1^L_a_ energy level shifts largely downward from that of pyrene and approaches the ^1^L_b_ energy level. Although the energetic ordering of the ^1^L_a_ and ^1^L_b_ states is unchanged, their energy levels are quite close to each other. The calculated ^1^L_a_ oscillator strength of 1,6-DPPy is larger than that of pyrene, which results in the ordering of pyrene < 1,6-DPPy < TPPy.

The TDDFT calculations also predict a downward shift of the ^1^L_a_ energy level without significant change of the ^1^L_b_ energy level. However, in contrary to the GMCQDPT results, the TDDFT calculations suggest that the ^1^L_a_ state is the lowest singlet excited state. As mentioned above, the ^1^L_b_ excitation energy of pyrene is significantly overestimated by the TDDFT calculations. The ^1^L_a_ energy level calculated using TD-B3LYP is estimated to be lower than the ^1^L_b_ energy level even for non-substituted pyrene. The gap between ^1^L_a_ and ^1^L_b_ is extremely underestimated using TD-CAM-B3LYP and TD-ωB97XD; because of this imbalanced alignment of the ^1^L_a_ and ^1^L_b_ energy levels for non-substituted pyrene, the energetic ordering of the ^1^L_a_ and ^1^L_b_ states is easily inverted through the downward shift of the ^1^L_a_ energy level. Meanwhile, the TDDFT calculations suggest an increase of the ^1^L_a_ oscillator strength through the diphenyl substitution as well as the GMCQDPT calculations. The oscillator strengths of the ^1^L_b_ state are much smaller than those of the ^1^L_a_ state, as with the cases of pyrene and TPPy. Yet, 1,6-DPPy exhibits the largest ^1^L_b_ oscillator strength among the molecules calculated here.

#### 3.1.4. 2,7-DPPy

In the results of GMCQDPT with MRX(4), the excitation energies of 2,7-DPPy only slightly decrease from those of pyrene for both ^1^L_a_ and ^1^L_b_. Consequently, the energy gap between the ^1^L_a_ and ^1^L_b_ states (0.34 eV) is only slightly decreased from the value in pyrene (0.44 eV). Contrary to the other two derivatives, 2,7-DPPy exhibits a decreased ^1^L_a_ oscillator strength compared to pyrene. Thus, the influence of phenyl substituents is dependent not only on the excited state but also on the substitution position.

The results of TDDFT calculations show a different behavior from the GMCQDPT results; the ^1^L_b_ energy level is more largely shifted downward without significant change of the ^1^L_a_ energy level. The behavior is also in contrast to the case of 1,6-DPPy which exhibits a largely downward shift of the ^1^L_a_ energy level without a major change in the ^1^L_b_ energy level. Meanwhile, a decrease of the ^1^L_a_ oscillator strength through diphenyl substitution is predicted using TDDFT, as with GMCQPDT.

### 3.2. Discussion

#### 3.2.1. Dependence on the reference space

**Fig. 3:**
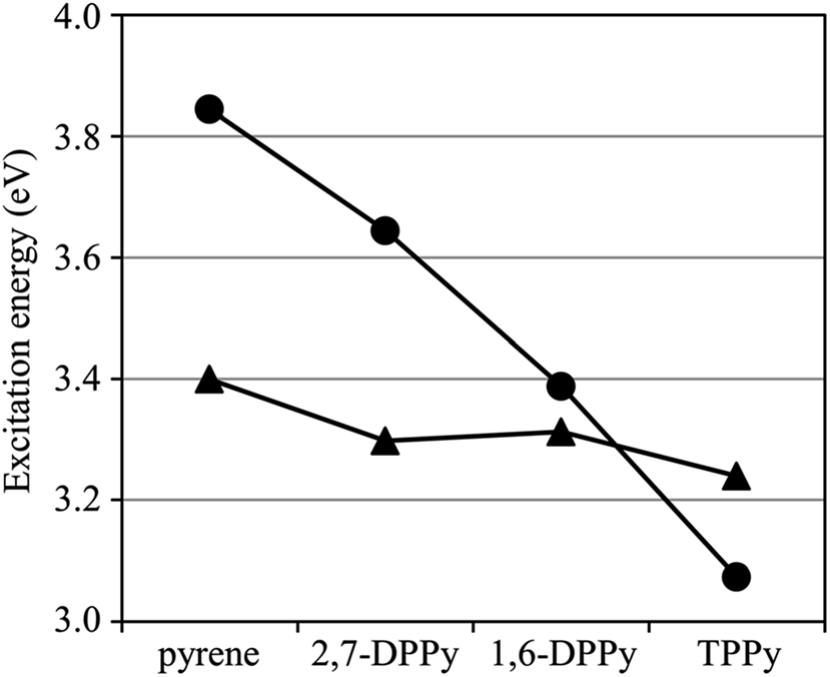
Excitation energies of the ^1^L_a_ (●) and ^1^L_b_ (▲) states calculated using GMCQDPT with MRX(4).

The excitation energies calculated using GMCQDPT with MRX(4) are plotted in Fig. 3 as a visualization of the energy level shifts of the ^1^L_a_ and ^1^L_b_ states through phenyl substitutions. The phenyl groups at 2,7-positions have less impact on the excitation energies. In contrast, the ^1^L_a_ energy level shifts largely downward in 1,6-DPPy without a significant change of the ^1^L_b_ energy level. The further downward shift of the ^1^L_a_ energy level causes the energetic ordering of the ^1^L_a_ and ^1^L_b_ states in TPPy to be inverted from that in pyrene. Therefore, the *ab initio* calculation results corroborate the ^1^L_a_–^1^L_b_ inversion that was hypothesized based on the experimentally-observed photophysical properties and supported by semi-empirical calculations.^68,69,72^ The results indicate that the fluorescence emission of TPPy has an ^1^L_a_ parentage, which is consistent with the high *q*_F_ and short *τ* of TPPy with respect to pyrene. Let us first discuss the dependence of the calculation results on the reference space.

The accuracy of the MRPT calculation reveals a strong dependence on the reference configuration space. Therefore, the requirements for the reference space to achieve reliable calculations can be determined from these results. The excitation energies with MCQDPT are highly dependent on the reference CAS, whereas those with GMCQDPT vary only in a narrow range. Thus, the calculation accuracy is sensitive to the number of active orbitals. There is no guarantee that MRPT with less active orbitals will be successful, even though its reference space is the CAS. In contrast, the incorporation of multiply-excited configurations into the reference space leads to a systematic improvement within the framework of a full π valence reference space. RASPT2 calculations by Nenov *et al.* implied that the reference space with incorporation of up to quadruple excitations is necessary to compute the excitation energies of pyrene with high accuracy.^101^ It should be noted that involving quadruply-excited configurations is also critically important for prediction of the ^1^L_a_–^1^L_b_ inversion.

The improvement of the calculation accuracy through the expansion of the reference space implies a significant multi-configuration character of these excited states. Therefore, the main configurations of the excited states in the MCSCF wave functions and their weights were analyzed (Table 2). The total weights of the main configurations for ^1^L_a_ and ^1^L_b_, denoted by *σ*_a_ and *σ*_b_, are also presented in Table 2. A smaller *σ* value indicates a greater contribution of the electron configurations other than the main configurations. Therefore, the *σ* value is correlated with the degree of the multi-configuration character of the excited state. The weights of the main configurations are relatively reduced as the contributions of more electron configurations are incorporated; therefore, the *σ* values are decreased with expansion of the reference space. Let us review the results obtained for pyrene with MRX(*n*); the *σ*_b_ values are generally smaller than the *σ*_a_ values and more largely varied depending on the reference space. Such behavior can be attributed to the significant multi-configuration character of ^1^L_b_ compared to ^1^L_a_, which is consistent with a high dependence of the calculated ^1^L_b_ excitation energy on the reference space (Table 1). The calculations with MRX(4) give the smallest *σ* values among the examined reference spaces for both L_a_ and L_b_; this is expected to be more suitable to deal with the complexity of these excited states. Although the *σ* values with CAS have similar trends to that with MRX(*n*), their fluctuations with the reference space are more pronounced and the values are generally larger than those with MRX(4). These results suggest that multi-configuration character is only insufficiently incorporated with these CAS references, even at the maximum CAS(12πe, 12πo). The *σ* values and their trends for TPPy, 1,6-DPPy and 2,7-DPPy are quite similar to those of pyrene, which indicates a comparable degree of the multi-configuration character of their excited states.

**Table 2:** Main configurations of the excited states and their weights. HOMO and LUMO are denoted as H and L, respectively. The values are rounded off to four decimal places

Molecule	Method	Reference space	^1^L_a_			^1^L_b_		
			H → L	H−1 → L+1	*σ*_a_	H → L+1	H−1 → L	*σ*_b_
Pyrene	GMCQDPT	MRX(2)	0.7295	0.1563	0.8858	0.4089	0.3971	0.8060
		MRX(3)	0.6808	0.1458	0.8265	0.3775	0.3594	0.7369
		MRX(4)	0.6577	0.1185	0.7762	0.3318	0.3267	0.6586
	MCQDPT	CAS(4πe, 4πo)	0.7906	0.2024	0.9930	0.5465	0.4451	0.9916
		CAS(8πe, 8πo)	0.7900	0.1163	0.9063	0.4259	0.3996	0.8255
		CAS(12πe, 12πo)	0.7370	0.0818	0.8188	0.3550	0.3605	0.7154
								
TPPy	GMCQDPT	MRX(2)	0.7699	0.1189	0.8888	0.4247	0.3829	0.8076
		MRX(3)	0.7250	0.1033	0.8283	0.3901	0.3487	0.7388
		MRX(4)	0.6923	0.0866	0.7789	0.3405	0.3195	0.6600
	MCQDPT	CAS(4πe, 4πo)	0.8638	0.1270	0.9907	0.5879	0.4040	0.9918
		CAS(8πe, 8πo)	0.8388	0.0680	0.9068	0.4377	0.3716	0.8093
		CAS(12πe, 12πo)	0.7730	0.0478	0.8208	0.3563	0.3415	0.6978
								
1,6-DPPy	GMCQDPT	MRX(4)	0.6330	0.0947	0.7277	0.3186	0.3040	0.6226
								
2,7-DPPy	GMCQDPT	MRX(4)	0.6238	0.1465	0.7703	0.3301	0.3285	0.6587

#### 3.2.2. Effects of phenyl groups on the excitation energies

We move on to the discussion on the effects of phenyl substitutions. The *σ* values analyzed above indicate that the ^1^L_a_ and ^1^L_b_ excited states have considerable multi-configuration characters. Nevertheless, the main configurations of these states still have large weights (Table 2), suggesting that the effects of phenyl groups might be essentially understood based on the molecular orbitals which are relevant to the main configurations: HOMO−1, HOMO, LUMO and LUMO+1. In this regard, the correlation between these molecular orbitals and the calculation results is explored.

The GMCSCF wave function with MRX(*n*) was constructed in terms of a linear combination of electron configurations and the GMCSCF calculations were carried out with a state-averaging scheme. Consequently, the GMCSCF orbital energies do not have clear physical meanings. The orbital energies obtained using the DFT calculations are dependent on the exchange-correlation functionals. Therefore, we analyzed the Hartree–Fock orbital energies collected in Table 3 where the energy gap between the p and q orbitals is denoted by *Δ*_p/q_; the energy shifts from the corresponding value of pyrene are shown in parentheses. The molecular orbitals obtained from the Hartree–Fock calculations are shown in Fig. S4.† In TPPy and 1,6-DPPy, the HOMO and LUMO energy levels are largely shifted compared to the HOMO−1 and LUMO+1 levels; the HOMO energy level is shifted upward, whereas the LUMO energy level is shifted downward. In 2,7-DPPy, in contrast, the HOMO−1 and LUMO+1 energy levels of 2,7-DPPy are largely shifted without significant changes of HOMO and LUMO energies. These variations of the orbital energies are consistent with the orbital distributions shown in Fig. 2 and S4.† The HOMO and LUMO of pyrene have large coefficients at positions 1, 3, 6, and 8; therefore, these orbitals are sensitive to phenyl substitution at these positions. In contrast, the HOMO−1 and LUMO+1 have large coefficients at positions 2 and 7; the phenyl substitution at positions 2 and 7 has a large impact on these orbitals. The electronic interactions between pyrene moieties and phenyl substituents can be also visually confirmed in the molecular orbitals of pyrene derivatives; the HOMO and LUMO of TPPy and 1,6-DPPy are partially extended to the phenyl substituents, whereas HOMO−1 and LUMO+1 are extended in 2,7-DPPy. The orbital extensions are more noticeable in the Hartree–Fock orbitals (Fig. S4†). As a result of the energetic changes of the orbitals, the *Δ*_HOMO/LUMO_ values are in the order of pyrene ≈ 2,7-DPPy > 1,6-DPPy > TPPy, which is in good agreement with that of the ^1^L_a_ excitation energies obtained using GMCQDPT with MRX(4) (Table 1). Although the *Δ*_HOMO/LUMO_ value of 2,7-DPPy is almost the same as that with pyrene, *Δ*_HOMO−1/LUMO+1_ of 2,7-DPPy is much smaller than that of pyrene. The HOMO−1 → LUMO+1 single excitation is another main configuration of the ^1^L_a_ states; therefore, a reduced *Δ*_HOMO−1/LUMO+1_ could be responsible for the slightly decreased ^1^L_a_ excitation energy. A similar correlation between the *Δ* values and excitation energies can be also found for the TDDFT results (Table 1). Thus, as for ^1^L_a_, the calculated excitation energies are well correlated with the *Δ* values related to the main configurations.

**Table 3:** Molecular orbital energies calculated using the Hartree–Fock method (eV)^a^

Orbital	Py	TPPy	1,6-DPPy	2,7-DPPy
LUMO+1	+2.58	+2.45	+2.50	+1.93
		(−0.13)	(−0.08)	(−0.65)
LUMO	+1.62	+1.32	+1.46	+1.55
		(−0.30)	(−0.16)	(−0.07)
HOMO	−7.03	−6.64	−6.82	−7.12
		(+0.39)	(+0.21)	(−0.09)
HOMO−1	−8.07	−7.95	−8.04	−7.48
		(+0.12)	(+0.03)	(+0.59)
*Δ*_HOMO/LUMO_	+8.65	+7.96	+8.28	+8.67
		(−0.69)	(−0.37)	(+0.02)
*Δ*_HOMO−1/LUMO+1_	+10.65	+10.40	+10.54	+9.41
		(−0.25)	(−0.11)	(−1.24)
*Δ*_HOMO/LUMO+1_	+9.61	+9.09	+9.32	+9.05
		(−0.52)	(−0.29)	(−0.56)
*Δ*_HOMO−1/LUMO_	+9.69	+9.27	+9.50	+9.03
		(−0.42)	(−0.19)	(−0.66)

a Values in parentheses are variations from the corresponding orbital energy of pyrene.

The calculated *Δ*_HOMO/LUMO+1_ and *Δ*_HOMO−1/LUMO_ values of phenyl substituted derivatives are lower than those of pyrene, which is consistent with their lower ^1^L_b_ excitation energies obtained using GMCQDPT with MRX(4). The fluctuations of the *Δ*_HOMO/LUMO+1_ and *Δ*_HOMO−1/LUMO_ values are rather milder than those of the *Δ*_HOMO/LUMO_ and *Δ*_HOMO−1/LUMO+1_ values. This behavior is also consistent with the insensitivity of the ^1^L_b_ excitation energies to phenyl substitution compared to the ^1^L_a_ excitation energies, which results in a reduction of the ^1^L_a_–^1^L_b_ gap in 1,6-DPPy and the ^1^L_a_–^1^L_b_ inversion in TPPy (Fig. 3). However, the *Δ*_HOMO/LUMO+1_ and *Δ*_HOMO−1/LUMO_ values are in the order of pyrene > 1,6-DPPy > TPPy > 2,7-DPPy, which is not in agreement with the ordering of the ^1^L_b_ excitation energies with GMCQDPT: pyrene > 1,6-DPPy ≈ 2,7-DPPy > TPPy (Table 1). The ^1^L_b_ state has significant multi-configuration character, as suggested from the less *σ* values (Table 2), which could be responsible for the weak correlation between the *Δ* values and the ^1^L_b_ excitation energies, and also for smaller fluctuations of the ^1^L_b_ excitation energies. In contrast to the GMCQDPT results, the ^1^L_b_ excitation energies with TDDFT exhibit a stronger correlation with the *Δ*_HOMO/LUMO+1_ and *Δ*_HOMO−1/LUMO_ values; these values are in the same order: pyrene > 1,6-DPPy > TPPy > 2,7-DPPy. Since TDDFT is a one-particle theory, the excitation energies obtained using TDDFT are susceptible to the energies of one-electron orbitals. Instead, the ^1^L_b_ excitation energy tends to be overestimated because of insufficient incorporation of multi-configuration character.

#### 3.2.3. Effects of phenyl groups on the oscillator strengths

The excitation energies are reasonably correlated with the *Δ* values, suggesting that the ^1^L_a_ and ^1^L_b_ states are primarily characterized by their main configurations. Therefore, the effects of phenyl substitution on the oscillator strength could be also understood based on the orbitals related to the main configurations. As for allowed transitions, spatial expansion of the relevant molecular orbitals generally leads to an increased oscillator strength through enlargement of the transition dipole moment. The HOMO and LUMO are spatially extended to four phenyl groups in TPPy and two phenyl groups in 1,6-DPPy (Fig. 2). Therefore, the calculated ^1^L_a_ oscillator strengths are in the order of TPPy > 1,6-DPPy > pyrene. In contrast, the calculated ^1^L_a_ oscillator strength of 2,7-DPPy is decreased from that of pyrene. The mechanism can also be understood based on the main configurations.^129,132^ A linear combination of HOMO → LUMO and HOMO−1 → LUMO+1 singly excited configurations results in ^1^L_a_ as a lower-energy component and ^1^B_a_ as a higher-energy component.^129^ The transition moments derived from these main configurations are counteracted with each other in ^1^L_a_, whereas they are reinforced in ^1^B_a_. The weights of these configurations are different; therefore, a transition moment still remains after their mutual cancellation, and the ^1^L_a_ state can thus give a visible absorption band. The difference in weights between these main configurations of 2,7-DPPy is reduced from that of pyrene (Table 2). The weight of the HOMO−1 → LUMO+1 excited configuration of 2,7-DPPy is specifically increased from that of pyrene, while that of the HOMO → LUMO excited configuration is decreased. The origin of such behavior is a reduced *Δ*_HOMO−1/LUMO+1_ without a major change in *Δ*_HOMO/LUMO_, so that the weight of the HOMO−1 → LUMO+1 excitation is relatively increased. In addition, the transition moment originated from the HOMO−1 → LUMO+1 single excitation is enlarged because these orbitals are partially extended to phenyl groups at positions 2 and 7. Therefore, the transition moments derived from these configurations are largely canceled by each other, which results in a decreased oscillator strength. Qiao *et al.* measured the UV/Vis absorption spectra of pyrene and its 2,7-substituted derivatives in dichloromethane.^121^ The results showed that 2,7-DPPy exhibited an absorption band with *λ*_max_ of 342 nm and this band is expected to have parentage from ^1^L_a_ of pyrene. The molar extinction coefficient is slightly smaller than that of the pyrene ^1^L_a_ absorption band. The present calculation results are consistent with the experimental observations. The linear combination of HOMO → LUMO+1 and HOMO−1 → LUMO singly excited configurations results in ^1^L_b_ as a lower-energy component and ^1^B_b_ as a higher-energy component.^129^ Therefore, the transition moments derived from these main configurations are counteracted each other in ^1^L_b_, whereas they are reinforced in ^1^B_b_. These configurations have almost the same weights in ^1^L_b_ (Table 2); therefore, the transition dipole moments are almost completely canceled, which results in its negligibly small oscillator strength. The cancellation is slightly suppressed in 1,6-DPPy because of its lower symmetry. Consequently, the ^1^L_b_ oscillator strength of 1,6-DPPy is high when compared to pyrene and other derivatives with higher symmetries.

The results of the TDDFT calculations are similar to those obtained from the GMCQDPT calculations; the calculated ^1^L_a_ oscillator strengths are in the order of TPPy > 1,6-DPPy > pyrene; the calculated ^1^L_b_ oscillator strengths are nearly zero except for 1,6-DPPy. Overall, the effects of substituents on the ^1^L_a_ excitation energy, the ^1^L_a_ and ^1^L_b_ oscillator strengths can be reasonably evaluated using TDDFT; however, the ^1^L_b_ excitation energy cannot be accurately predicted; in the worse cases, even the energetic ordering is incorrectly predicted. Thus, a multi-reference treatment is essential for an accurate calculation of the ^1^L_b_ excitation energy, the ordering of the ^1^L_a_ and ^1^L_b_ states, and the energy gap between these states.

## 4. Conclusion

The absorption and fluorescence emission behavior of pyrene derivatives is characterized by the low-lying excited states derived from ^1^L_a_ and ^1^L_b_ of pyrene. In this study, pyrene, TPPy, 1,6-DPPy, and 2,7-DPPy as typical examples were calculated using MRPT methods to explore a dependable computational scheme for these excited states. When all valence π and π* orbitals on the pyrene moiety were incorporated into the reference space, the calculated ^1^L_a_ and ^1^L_b_ excitation energies of pyrene were in good agreement with the experimental values. The fluorescence emission of TPPy was predicted to have pyrene ^1^L_a_ parentage, which theoretically corroborates the experimental observations. A reference space involving singly, doubly, triply, and quadruply excited configurations was required to predict the ^1^L_a_–^1^L_b_ inversion through tetraphenyl substitution. MRPT calculations with smaller reference spaces and single-reference theory calculations exhibit inconsistencies with these results, which suggests that adequate treatment of the multi-configuration character is essential. The effect of phenyl substitution was dependent not only on the excited state but also on the substitution position. The detailed mechanism was clarified by examination of the MCSCF wave functions. These calculations were successfully conducted at a tractable computational cost by the adoption of GMCQDPT to enable the handling of general types of reference configuration space.

## Conflicts of interest

There are no conflicts of interest to declare.

## Supplementary Material

RA-010-C9RA10483F-s001
