# Supplementary material for: Ab initio study on the excited states of pyrene and its derivatives using multi-reference perturbation theory methods
Source: RSC Adv. 2020 Mar 31;10(22):12988–98. doi: 10.1039/c9ra10483f (PMC9051409; doi:10.1039/c9ra10483f)
Supplement: RA-010-C9RA10483F-s001 [file RA-010-C9RA10483F-s001.pdf]

Electronic Supplementary Material (ESI) for RSC Advances.
This journal is © The Royal Society of Chemistry 2020

Supporting Information

Ab initio study on the excited states of pyrene and its derivatives using multi-reference perturbation theory methods

Soichi Shirai* and Shinji Inagaki

* Author to whom correspondence should be addressed.

E-mail: shirai@mosk.tytlabs.co.jp

†Toyota Central R&D Laboratories, Inc., Nagakute, Aichi 480-1192, Japan

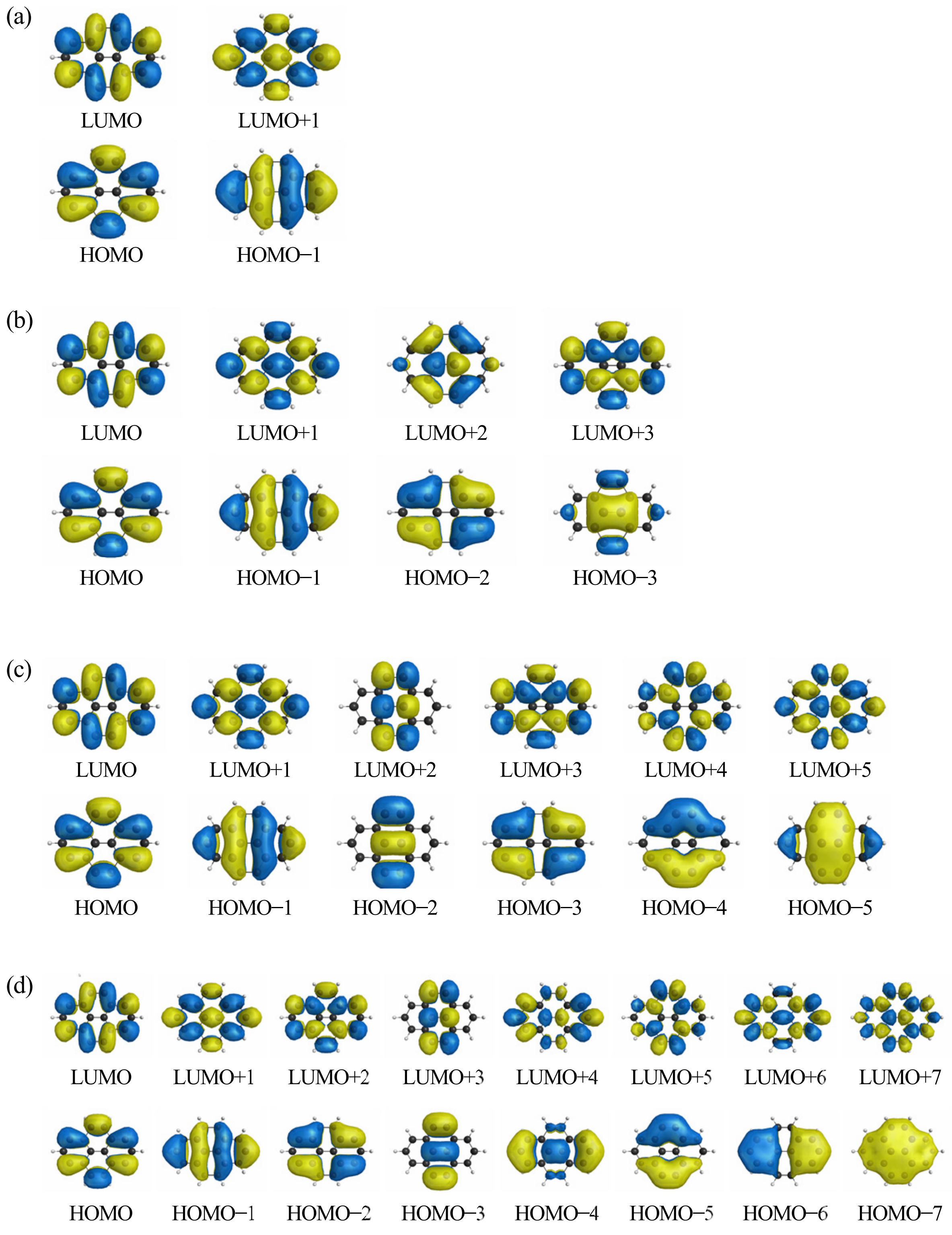

Fig. S1 Natural orbitals of pyrene from MCSCF wave functions:
(a) CAS(4πe, 4πo), (b) CAS(8πe, 8πo), (c) CAS(12πe, 12πo) and (d) MRX(4).

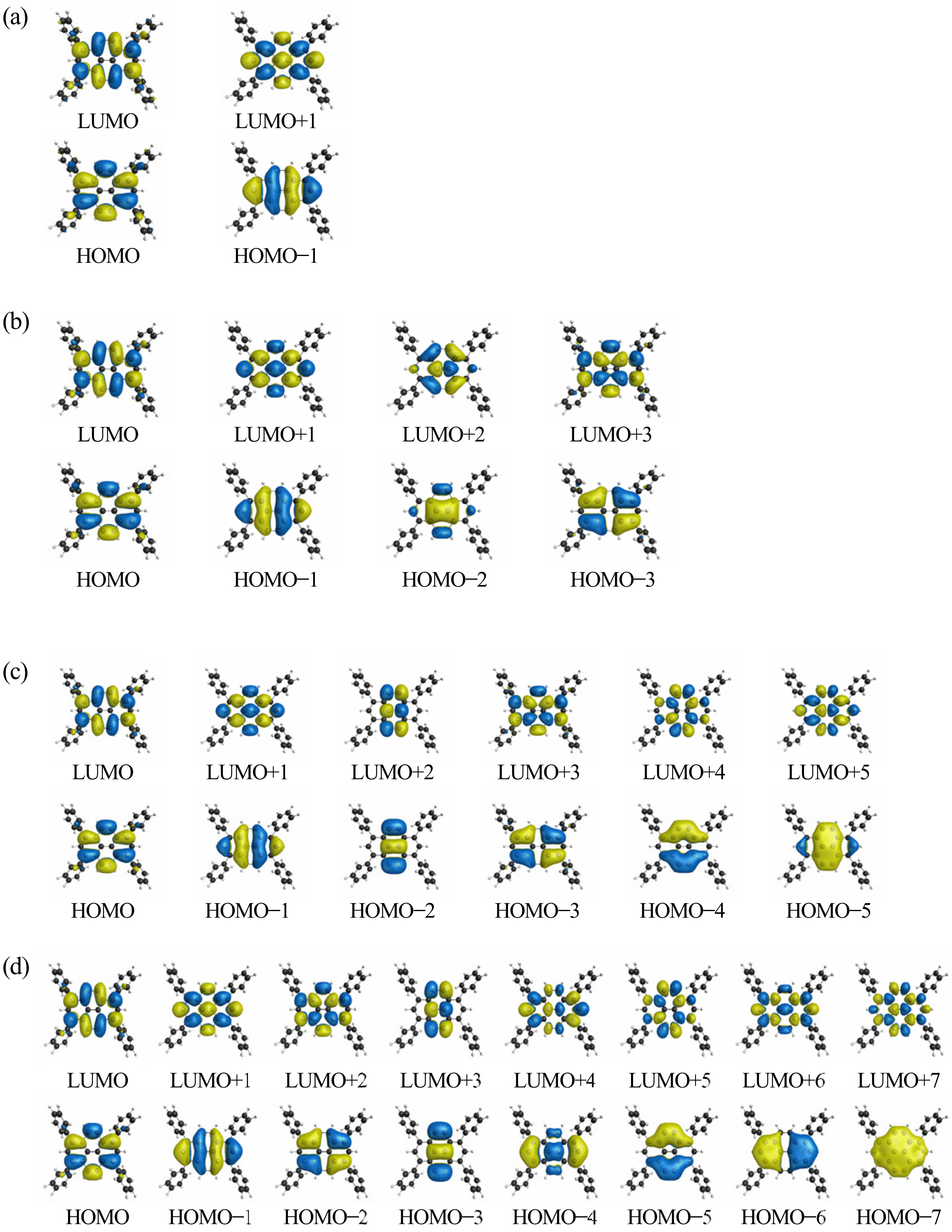

Fig. S2 Natural orbitals of 1,3,6,8-tetraphenylpyrene (TPPy) from MCSCF wave functions: (a) CAS(4*π*e, 4*π*o), (b) CAS(8*π*e, 8*π*o), (c) CAS(12*π*e, 12*π*o) and (d) MRX(4).

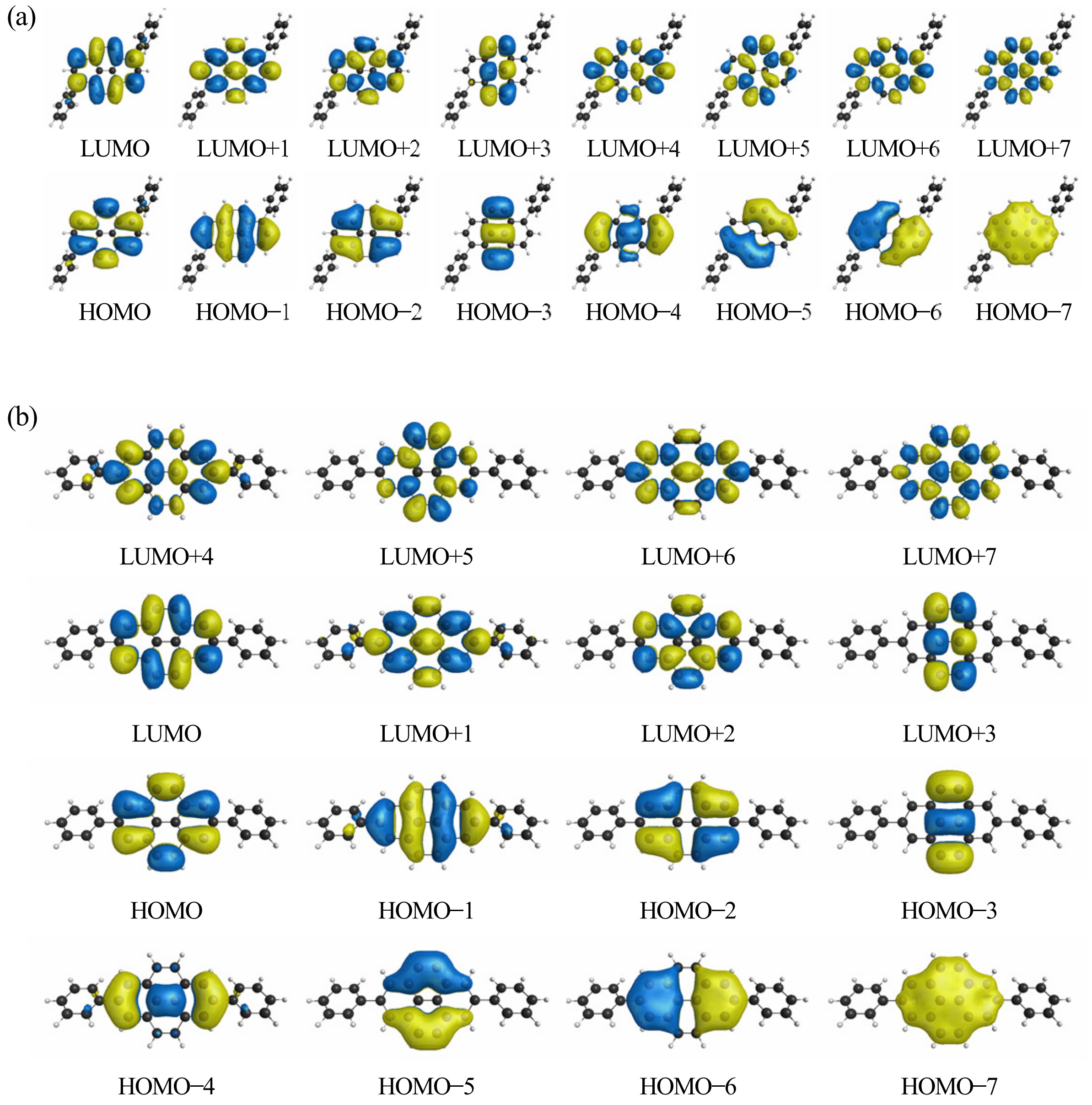

Fig. S3 Natural orbitals of (a) 1,6-diphenylpyrene (1,6-DPPy) and (b) 2,7-diphenylpyrene (2,7-DPPy) from MCSCF wave functions with MRX(4) configuration space.

Table S1. Calculated electronic total energies and excitation energies.

molecule	method	reference space	electronic state total energy			excitation energy	
			ground state	^1L_a	^1L_b	^1L_a	^1L_b
pyrene	GMCSCF	MRX(2)	−611.963398	−611.683908	−611.732426	7.61	6.28
		MRX(3)	−611.969412	−611.773601	−611.827047	5.33	3.87
		MRX(4)	−611.998334	−611.786239	−611.854412	5.77	3.92
	CASSCF	CAS(4πe, 4πo)	−611.826258	−611.630826	−611.633173	5.32	5.25
		CAS(8πe, 8πo)	−611.873724	−611.673541	−611.698314	5.45	4.77
		CAS(12πe, 12πo)	−611.938240	−611.733420	−611.773669	5.57	4.48
	GMCQDPT	MRX(2)	−613.895808	−613.757849	−613.779477	3.75	3.17
		MRX(3)	−613.898739	−613.758249	−613.777274	3.82	3.31
		MRX(4)	−613.904548	−613.763264	−613.779616	3.84	3.40
	MCQDPT	CAS(4πe, 4πo)	−613.888464	−613.758129	−613.784435	3.55	2.83
		CAS(8πe, 8πo)	−613.895007	−613.757183	−613.775339	3.75	3.26
		CAS(12πe, 12πo)	−613.912130	−613.771275	−613.764713	3.83	4.01
	EOM-CCSD		−613.927251	−613.763560	−613.786867	4.45	3.82
TPPy	GMCSCF	MRX(2)	−1530.208096	−1529.939689	−1529.982544	7.30	6.14
		MRX(3)	−1530.214345	−1530.028781	−1530.075887	5.05	3.77
		MRX(4)	−1530.243303	−1530.040528	−1530.102935	5.52	3.82
	CASSCF	CAS(4πe, 4πo)	−1530.072126	−1529.887052	−1529.892707	5.04	4.88
		CAS(8πe, 8πo)	−1530.119736	−1529.934120	−1529.950080	5.05	4.62
		CAS(12πe, 12πo)	−1530.183896	−1529.992171	−1530.023859	5.22	4.35
	GMCQDPT	MRX(2)	−1535.272512	−1535.158154	−1535.163555	3.11	2.96
		MRX(3)	−1535.274668	−1535.154509	−1535.159934	3.27	3.12
		MRX(4)	−1535.280775	−1535.167838	−1535.161723	3.07	3.24
	MCQDPT	CAS(4πe, 4πo)	−1535.277130	−1535.169965	−1535.181531	2.92	2.60
		CAS(8πe, 8πo)	−1535.306796	−1535.191025	−1535.195163	3.15	3.04
		CAS(12πe, 12πo)	−1535.368049	−1535.232284	−1535.236305	3.69	3.58
	EOM-CCSD		−1535.391457	−1535.247783	−1535.257238	3.91	3.65
1,6-DPPy	GMCSCF	MRX(4)	−1071.121009	−1070.913546	−1070.978846	5.65	3.87
	GMCQDPT	MRX(4)	−1074.592114	−1074.467613	−1074.470366	3.39	3.31
2,7-DPPy	GMCSCF	MRX(4)	−1071.128369	−1070.917149	−1070.987240	5.75	3.84
	GMCQDPT	MRX(4)	−1074.592383	−1074.458462	−1074.471182	3.64	3.30

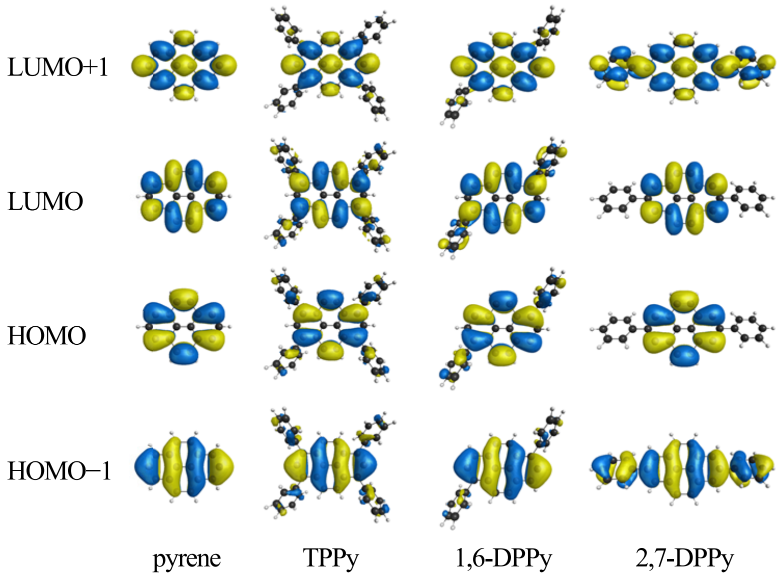

Fig. S4 Molecular orbitals of pyrene, TPPy, 1,6-DPPy, and 2,7-DPPy obtained from the Hartee-Fock calculations.
